# Supplementary material for: Promising M2CO2/MoX2 (M = Hf, Zr; X = S, Se, Te) Heterostructures for Multifunctional Solar Energy Applications
Source: Molecules. 2023 Apr 17;28(8):3525. doi: 10.3390/molecules28083525 (PMC10146659; doi:10.3390/molecules28083525)
Supplement: Supplementary file 1 [file molecules-28-03525-s001.zip › molecules-2331595-supplementary.pdf]

molecules
MDPI

Electronic Supplementary Material for

Promising M_2CO_2/MoX_2 (M=Hf, Zr; X=S, Se, Te) Heterostructures for Multifunctional Solar Energy Applications

Jiansen Wen [1], Qi Cai [1], Rui Xiong [1], Zhou Cui [1], Yinggan Zhang [2], Zhihan He [1], Junchao Liu [1], Maohua Lin [3], Cuilian Wen [1], Bo Wu [1,*] and Baisheng Sa [1,*]

[1] Multiscale Computational Materials Facility, and Key Laboratory of Eco-Materials Advanced Technology, College of Materials Science and Engineering, Fuzhou University, Fuzhou 350100, China
[2] College of Materials, Xiamen University, Xiamen 361005, China
[3] Department of Ocean and Mechanical Engineering, Florida Atlantic University, Boca Raton, FL 33431, USA
* Correspondence: wubo@fzu.edu.cn (B.W.); bssa@fzu.edu.cn (B.S.)

Table S1. The lattice constants *a* (Å), band gaps E_g (eV) and band gap types of M_2CO_2 (M = Zr, Hf) and MoX_2 (X = S, Se, Te) monolayers

System	Functional	*a*	E_g	Type	Reference
Zr_2CO_2	PBE	3.31	0.95		[1]
	PBE	3.31	0.87		[2]
	PBE	3.31			[3]
	DFT-D3	3.30	0.89	Indirect	This work
	OptB86b	3.31	0.98	Indirect	This work
	HSE06		1.71		[3]
	HSE06		1.59		[2]
	HSE06		1.68	Indirect	This work
Hf_2CO_2	PBE	3.27	1.00		[1]
	PBE	3.27	0.99		[2]
	PBE	3.27			[3]
	DFT-D3	3.25	0.93	Indirect	This work
	OptB86b	3.27	1.17	Indirect	This work
	HSE06		1.68		[3]
	HSE06		1.70		[2]
	HSE06		1.69	Indirect	This work
MoS_2	DFT-D2	3.17	1.70		[4]
	PBE	3.18	1.68		[5]
	DFT-D3	3.16	1.76	Direct	This work
	OptB86b	3.18	1.67	Direct	This work
	HSE06		1.97		[4]
	HSE06		2.23	Direct	This work
$MoSe_2$	DFT-D3	3.30			[6]
	DFT-D2	3.32	1.43	Direct	[7]
	PBE	3.32	1.45		[5]
	DFT-D3	3.29	1.53	Direct	This work
	OptB86b	3.32	1.44	Direct	This work
	HSE06		1.98		[6]
	HSE06		2.00	Direct	This work
$MoTe_2$	DFT-D3	3.52			[8]
	PBE	3.55	1.08		[5]
	DFT-D3	3.52	1.17	Direct	This work
	OptB86b	3.56	1.07	Direct	This work
	HSE06		1.54	Direct	[8]
	HSE06		1.63	Direct	This work

Table S2. The total energy (eV) of different stacking configurations for M_2CO_2/MoX_2 heterostructures

Stacking configuration	I	II	III	IV	V	VI
Hf_2CO_2/MoS_2	-73.9222	-73.9317	-73.8583	-73.9192	-73.8604	-73.9285
$Hf_2CO_2/MoSe_2$	-72.2279	-72.2442	-72.1693	-72.2302	-72.1714	-72.2347
$Hf_2CO_2/MoTe_2$	-70.0721	-70.0879	-70.0020	-70.0896	-70.0025	-70.0727
Zr_2CO_2/MoS_2	-70.4593	-70.4702	-70.3918	-70.4549	-70.3946	-70.4675
$Zr_2CO_2/MoSe_2$	-68.8266	-68.8443	-68.7657	-68.8274	-68.7683	-68.8346
$Zr_2CO_2/MoTe_2$	-66.7241	-66.7569	-66.6711	-66.7374	-66.6741	-66.7357

Table S3. The lattice constants *a* (Å), interlayer distance *d* (Å), degree of lattice mismatch *K*, formation energy E_f (meV) and binding energy E_b (meV/Å^2) for the most stable configurations of M_2CO_2/MoX_2 heterostructures

System	*a*	*d*	*K*	E_f	E_b
Hf_2CO_2/MoS_2	3.23	2.72	2.7%	-212.294	-29.243
Hf_2CO_2/$MoSe_2$	3.26	2.82	1.2%	-268.752	-30.580
Hf_2CO_2/$MoTe_2$	3.32	3.05	7.9%	5.318	-35.552
Zr_2CO_2/MoS_2	3.26	2.70	4.1%	-159.852	-28.785
Zr_2CO_2/$MoSe_2$	3.30	2.79	0.1%	-277.978	-29.727
Zr_2CO_2/$MoTe_2$	3.36	2.96	6.5%	-71.028	-30.801

Table S4. The calculated band gap E_g (eV) of the most stable configurations for M_2CO_2/MoX_2 heterostructures

System	Functional	E_g
Hf_2CO_2/MoS_2	DFT-D3	0.68
	HSE06	1.35
Hf_2CO_2/$MoSe_2$	DFT-D3	0.97
	HSE06	1.64
Hf_2CO_2/$MoTe_2$	DFT-D3	0.34
	HSE06	0.66
Zr_2CO_2/MoS_2	DFT-D3	0.50
	HSE06	1.11
Zr_2CO_2/$MoSe_2$	DFT-D3	0.90
	HSE06	1.69
Zr_2CO_2/$MoTe_2$	DFT-D3	0.73
	HSE06	1.13

Table S5. The total charge transfer amounts between MoX_2 Q_{MoX_2} and M_2CO_2 $Q_{M_2CO_2}$ in the M_2CO_2/MoX_2 heterostructures

System	Q_{MoX_2}	$Q_{M_2CO_2}$
Hf_2CO_2/MoS_2	-0.0066	0.0066
Hf_2CO_2/$MoSe_2$	-0.0104	0.0104
Hf_2CO_2/$MoTe_2$	-0.0157	0.0157
Zr_2CO_2/MoS_2	0.0158	-0.0158
Zr_2CO_2/$MoSe_2$	-0.0094	0.0094
Zr_2CO_2/$MoTe_2$	-0.0197	0.0197

Table S6. The conduction band offset ΔE_c (eV), donor band gap E_g^d (eV), and calculated power conversion efficiency (PCE) η (%) of M_2CO_2/MoX_2 heterostructures for solar cell applications

System	ΔE_c	E_g^d	η
Hf_2CO_2/MoS_2	0.097	1.64	19.75
Hf_2CO_2/$MoSe_2$	0.628	2.08	9.64
Hf_2CO_2/$MoTe_2$	0.396	1.09	11.34
Zr_2CO_2/MoS_2	0.272	1.58	17.13
Zr_2CO_2/$MoSe_2$	0.433	1.95	12.42
Zr_2CO_2/$MoTe_2$	0.370	1.29	14.46

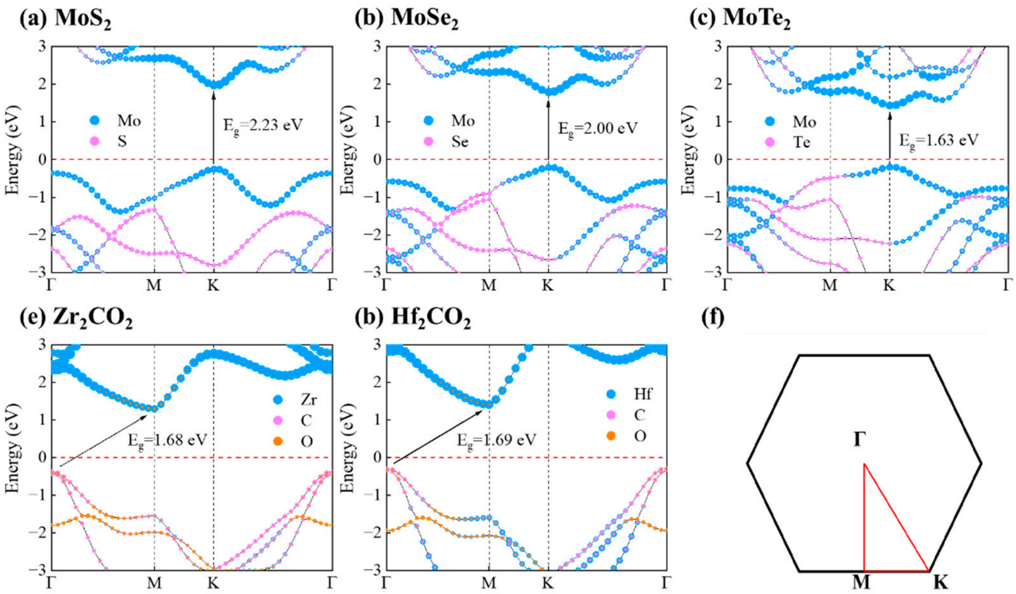

Figure S1. The projected band structure of (a) MoS_2, (b) $MoSe_2$, (c) $MoTe_2$, (d) Zr_2CO_2, (e) Hf_2CO_2 monolayers. (f) The first Brillouin zone and high symmetry points of the hexagonal 2D lattice.

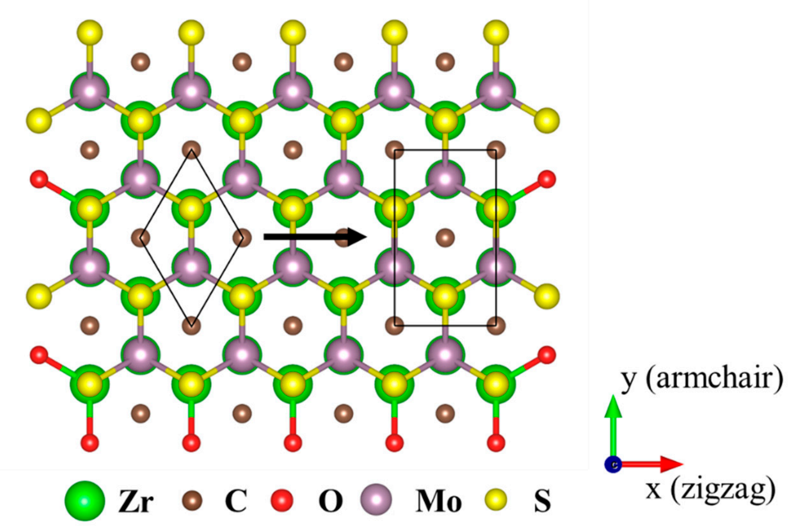

Figure S2. An orthorhombic lattice instead of the traditional hexagonal lattice was adopted to calculate the intrinsic responses to uniaxial strain in the Zr_2CO_2/MoS_2 heterostructure.

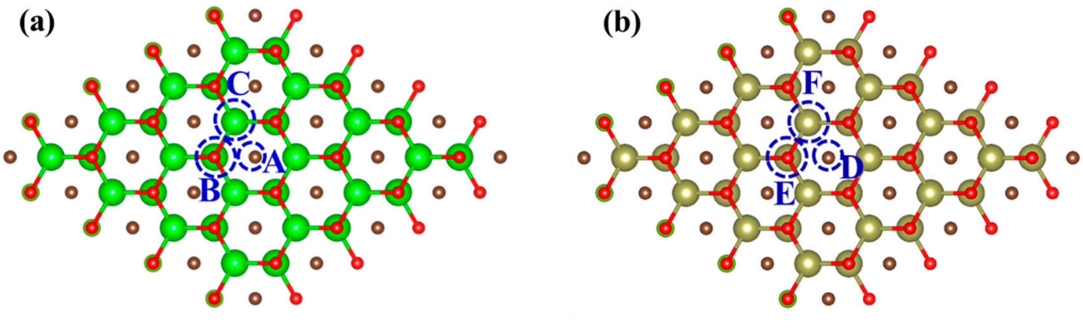

Figure S3. Different adsorption sites of the water molecule on the (a) Zr_2CO_2 and (b) Hf_2CO_2 surfaces of the $Zr_2CO_2/MoSe_2$ and $Hf_2CO_2/MoSe_2$ heterostructures.

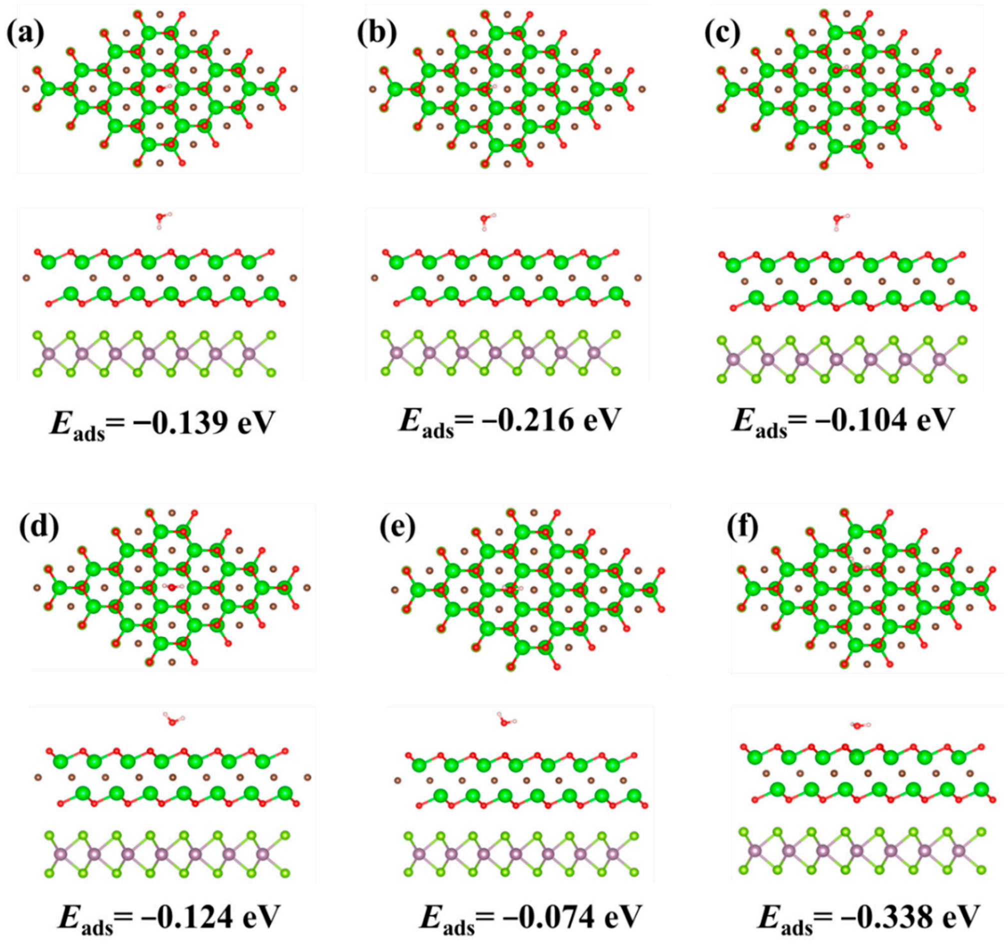

Figure S4. Optimized structures and adsorption energies of the water molecule with H as the adsorption atom at (a) A, (b) B, (c) C adsorption site and O as the adsorption atom at (d) A, (e) B, (f) C adsorption site on the Zr_2CO_2 surface of the $Zr_2CO_2/MoSe_2$ heterostructure.

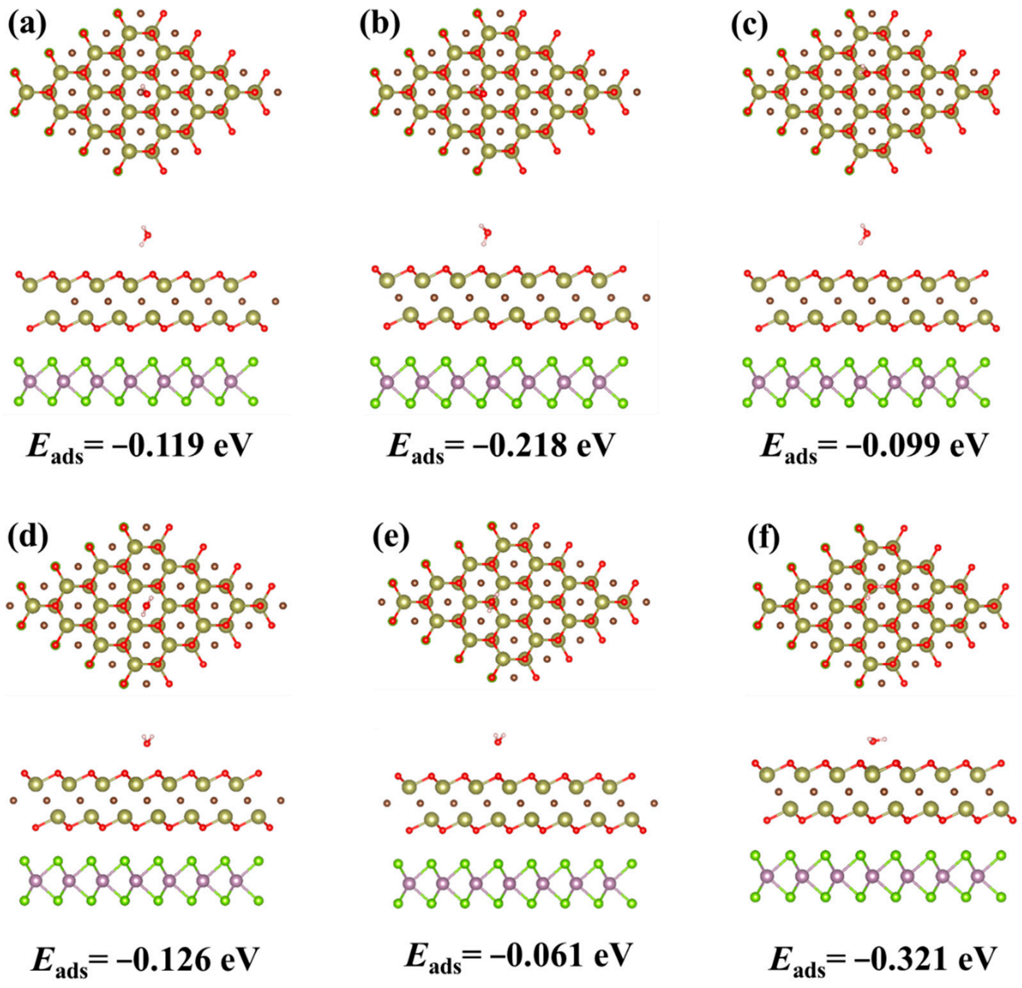

Figure S5. Optimized structures and adsorption energies of the water molecule with H as the adsorption atom at (a) D, (b) E, (c) F adsorption site and O as the adsorption atom at (d) D, (e) E, (f) F adsorption site on the Hf_2CO_2 surface of the $Hf_2CO_2/MoSe_2$ heterostructure.

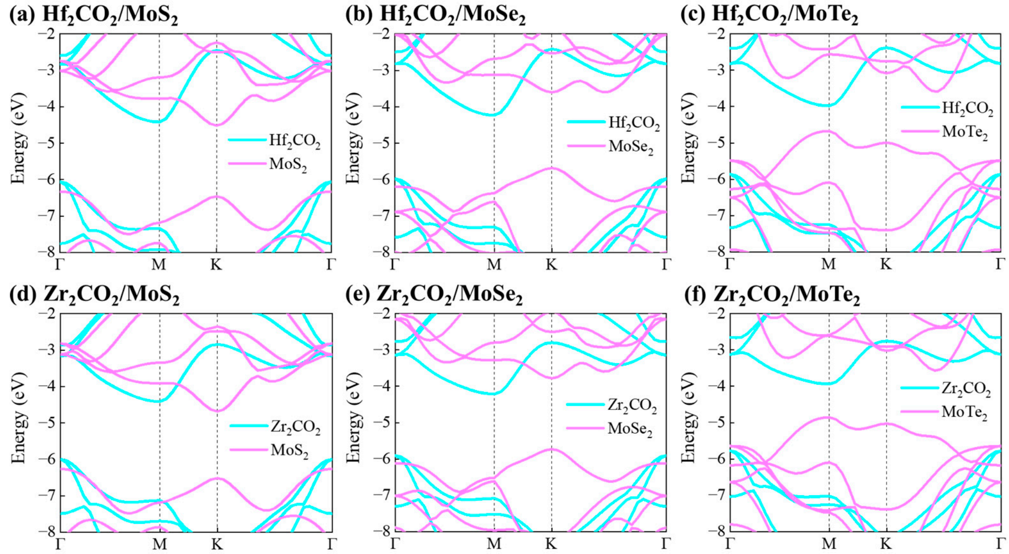

Figure S6. The HSE06 band structures of mutually independent monolayers fixed in the heterostructure lattices for (a) Hf_2CO_2/MoS_2, (b) $Hf2CO_2/MoSe_2$, (c) $Hf_2CO_2/MoTe_2$, (d) Zr_2CO_2/MoS_2, (e) $Zr_2CO_2/MoSe_2$ and (f) $Zr_2CO_2/MoTe_2$.

References

1. Gandi, A.N.; Alshareef, H.N.; Schwingenschlögl, U. Thermoelectric performance of the MXenes M_2CO_2 (M = Ti, Zr, or Hf). Chem. Mater. 2016, 28, 1647-1652. https://doi.org/10.1021/acs.chemmater.5b04257.
2. Munawar, M.; Idrees, M.; Ahmad, I.; Din, H.U.; Amin, B. Intriguing electronic, optical and photocatalytic performance of BSe, M_2CO_2 monolayers and BSe–M_2CO_2 (M = Ti, Zr, Hf) van der Waals heterostructures. RSC Adv. 2022, 12, 42-52. http://doi.org/10.1039/D1RA07569A.
3. Fu, C.; Li, X.; Luo, Q.; Yang, J. Two-dimensional multilayer M_2CO_2 (M = Sc, Zr, Hf) as photocatalysts for hydrogen production from water splitting: A first principles study. J. Mater. Chem. A 2017, 5, 24972-24980. http://doi.org/10.1039/C7TA08812D.
4. Xu, X.; Ge, X.; Liu, X.; Li, L.; Fu, K.; Dong, Y.; Meng, F.; Si, R.; Zhang, M. Two-dimensional M_2CO_2/MoS_2 (M = Ti, Zr and Hf) van der Waals heterostructures for overall water splitting: A density functional theory study. Ceram. Int. 2020, 46, 13377-13384. https://doi.org/10.1016/j.ceramint.2020.02.119.
5. Zhuang, H.L.; Hennig, R.G. Computational search for Single-Layer Transition-Metal dichalcogenide photocatalysts. The Journal of Physical Chemistry C 2013, 117, 20440-20445. https://doi.org/10.1021/jp405808a.
6. Xu, X.; Wu, X.; Tian, Z.; Zhang, M.; Li, L.; Zhang, J. Modulating the electronic structures and potential applications of Zr_2CO_2/MSe_2 (M = Mo, W) heterostructures by different stacking modes: A density functional theory calculation. Appl. Surf. Sci. 2022, 599, 154014. https://doi.org/10.1016/j.apsusc.2022.154014.
7. Li, X.; Cui, X.; Xing, C.; Cui, H.; Zhang, R. Strain-tunable electronic and optical properties of Zr_2CO_2 MXene and $MoSe_2$ van der Waals heterojunction: A first principles calculation. Appl. Surf. Sci. 2021, 548, 149249. https://doi.org/10.1016/j.apsusc.2021.149249.
8. Wang, B.; Wang, X.; Wang, P.; Kuang, A.; Zhou, T.; Yuan, H.; Chen, H. Bilayer $MoTe_2/XS_2$ (X = Hf, Sn, Zr) heterostructures with efficient carrier separation and light absorption for photocatalytic water splitting into hydrogen. Appl. Surf. Sci. 2021, 544, 148842. https://doi.org/10.1016/j.apsusc.2020.148842.

Disclaimer/Publisher's Note: The statements, opinions and data contained in all publications are solely those of the individual author(s) and contributor(s) and not of MDPI and/or the editor(s). MDPI and/or the editor(s) disclaim responsibility for any injury to people or property resulting from any ideas, methods, instructions or products referred to in the content.
